# Effects of Chronic Cannabidiol Treatment in the Rat Chronic Unpredictable Mild Stress Model of Depression

**DOI:** 10.3390/biom10050801

**Published:** 2020-05-22

**Authors:** Zsolt Gáll, Szidónia Farkas, Ákos Albert, Elek Ferencz, Szende Vancea, Melinda Urkon, Melinda Kolcsár

**Affiliations:** 1 Department of Pharmacology and Clinical Pharmacy, George Emil Palade University of Medicine, Pharmacy, Science, and Technology of Targu Mures, 540142 Târgu Mureș, Romania; farkasaszidonia@gmail.com (S.F.); albert_akos@yahoo.com (A.A.); urkonmelinda1@gmail.com (M.U.); melinda.kolcsar@umfst.ro (M.K.); 2 Department of Physical Chemistry, George Emil Palade University of Medicine, Pharmacy, Science, and Technology of Targu Mures, 540142 Târgu Mureș, Romania; elekferencz@yahoo.com (E.F.); vancsa.szende@gmail.com (S.V.)

**Keywords:** cannabidiol, depression, animal model, chronic mild stress, hair corticosterone

## Abstract

Several neuropharmacological actions of cannabidiol (CBD) due to the modulation of the endocannabinoid system as well as direct serotonergic and gamma-aminobutyric acidergic actions have recently been identified. The current study aimed to reveal the effect of a long-term CBD treatment in the chronic unpredictable mild stress (CUMS) model of depression. Adult male Wistar rats (n = 24) were exposed to various stressors on a daily basis in order to induce anhedonia and anxiety-like behaviors. CBD (10 mg/kg body weight) was administered by daily intraperitoneal injections for 28 days (n = 12). The effects of the treatment were assessed on body weight, sucrose preference, and exploratory and anxiety-related behavior in the open field (OF) and elevated plus maze (EPM) tests. Hair corticosterone was also assayed by liquid chromatography–mass spectrometry. At the end of the experiment, CBD-treated rats showed a higher rate of body weight gain (5.94% vs. 0.67%) and sucrose preference compared to controls. A significant increase in vertical exploration and a trend of increase in distance traveled in the OF test were observed in the CBD-treated group compared to the vehicle-treated group. The EPM test did not reveal any differences between the groups. Hair corticosterone levels increased in the CBD-treated group, while they decreased in controls compared to baseline (+36.01% vs. −45.91%). In conclusion, CBD exerted a prohedonic effect in rats subjected to CUMS, demonstrated by the increased sucrose preference after three weeks of treatment. The reversal of the effect of CUMS on hair corticosterone concentrations might also point toward an anxiolytic or antidepressant-like effect of CBD, but this needs further confirmation.

## 1. Introduction

Cannabidiol (CBD), the major non-psychotomimetic component of different *Cannabis* species, has been the subject of numerous preclinical and clinical studies, since the endocannabinoid system and its role in neuropsychiatric disorders were first recognized [1,2]. The anticonvulsant action of CBD in various childhood epilepsy syndromes (e.g., Dravet syndrome, Lennox–Gastaut syndrome) has been demonstrated, and now CBD is available as an ‘orphan medicine’ in the European Union and the United States [3,4,5]. It is also marketed in combination with Δ^9^-tetrahydrocannabinol, the product known as Sativex^®^, for spasticity and pain in multiple sclerosis. Furthermore, its use in veterinary practice was also recommended [6,7].

Due to its multiple mechanisms of action, CBD was proposed for the treatment of most psychiatric diseases characterized by increased anxiety [8]. Generalized social anxiety disorder, panic disorder, obsessive–compulsive disorder, and post-traumatic stress disorder have been comprehensively examined not only in animal models but also in clinical experiences [9,10,11,12,13]. Besides the abovementioned properties, CBD potential antipsychotic, neuroprotective [14], and antidepressant [15] effects have been studied lately.

Depression remains a leading neuropsychiatric condition causing one of the greatest economic burden [16]. Drugs acting on monoamine neurotransmission have dominated the treatment of depression for decades; however, a significant percentage of depressive patients show resistance to pharmacotherapy [17]. Recently, ketamine and its enantiomer *S*-ketamine have been rediscovered as rapid acting antidepressants with demonstrated efficiency in treatment-resistant depression [18,19]. CBD has also been reported to produce antidepressant-like effects after a single dose [15,20,21]. However, its chronic administration in animal models of depression has not been extensively studied.

The chronic unpredictable mild stress (CUMS) model is a widely used and well-established animal model of depression [22], whose supposed underlying mechanisms include stress-induced neural reorganization, neurotransmitter alterations in brain regions associated with depression, and microglial activation leading to altered hippocampal functions and inflammation [23,24,25]. This model is associated with anhedonia, increased anxiety-like behavior, and impaired function of the hypothalamic–pituitary–adrenocortical (HPA) system in rats [26]. Although it was designed to study the neurobiology of depression, its application in drug development and screening became a routine after conventional antidepressants were proved to reverse CUMS-induced effects. Moreover, several drugs shown to possess antidepressant activity in the CUMS model were confirmed to act similarly in humans [27]. The increased level of plasma corticosterone (CORT) has been used as a stress biomarker in rodents, reflecting the acute response to stress, but its values may vary depending on sampling conditions. An important aspect to consider is that the half-life of CORT in plasma is approximately 20 min, therefore, it is necessary to conduct repeated measurements of plasma CORT levels. On the other hand, chronic stress decreases the HPA system responsivity to acute stress, leading to decreased peak plasma CORT levels [28]. In particular, CUMS was demonstrated to induce hyporesponsiveness of the HPA axis in female rats, but basal plasma CORT levels were not influenced [25]. Hair CORT concentration was recently proposed as a tool to describe HPA activity over a longer period and as such it could serve to describe the chronic dysregulation of the HPA axis [29,30].

The main purposes of the current study were to assess the effects of long-term CBD administration in CUMS-induced depression, using behavioral tests and hair CORT level determination in rats, and to evaluate whether CBD could be an alternative for chronic therapy of depression or anxiety.

## 2. Materials and Methods

### 2.1. Animals

Experimentally naive, adult male Wistar rats (provided by the George Emil Palade University of Medicine, Pharmacy, Science, and Technology of Targu Mures, mean weight 387 ± 32 g) were included, which prior to the experiments, were subjected to 21-day habituation to single housing, handling, and standard environmental conditions (12 h light–dark cycle, 20 ± 2 °C temperature, 60% ± 10% humidity). Standard pelleted rodent chow and water were provided ad libitum, except during the deprivation periods within the CUMS protocol. Bodyweight was recorded once or twice weekly, depending on the application of food deprivation as a stress factor. The applied procedures were in accordance with European Directive 2010/63/EU and approved by the Ethics Committee for Scientific Research of the George Emil Palade University of Medicine, Pharmacy, Science, and Technology of Targu Mures (approval no. 8/2018).

This study was designed to evaluate the long-term effects of CBD in the CUMS model of depression. For this, a preliminary acute experiment was conducted to observe the behavioral effects of CBD at peak plasma concentration (approximately 1 h after a single dose administration) in non-stressed drug-naïve rats. The animals were randomly divided into two groups, a control group (received the vehicle intraperitoneally in a volume of 1 mL/kg, n = 8) and a CBD-treated group (administered intraperitoneally (i.p.) CBD 10 mg/kg, n = 8). The results of the acute experiment were used to calculate the required sample sizes for the chronic experiment using statistical power analysis. Based on this, 24 animals underwent the CUMS procedure after they were randomly divided into a control group (CUMS group received the vehicle i.p. in a volume of 1 ml/kg, n = 12) and a CBD-treated group (CUMS + CBD group, treated with daily i.p. CBD, n = 12). Both the control and the CBD-treated group underwent the same care and injection protocol and were evaluated equally. The third group of animals (sham, n = 12) was used to test the influence of isolation on hair CORT levels; animals of this group were administered daily i.p. injections of the vehicle but were not exposed to stress.

### 2.2. Drugs and Reagents

**Figure 1:**
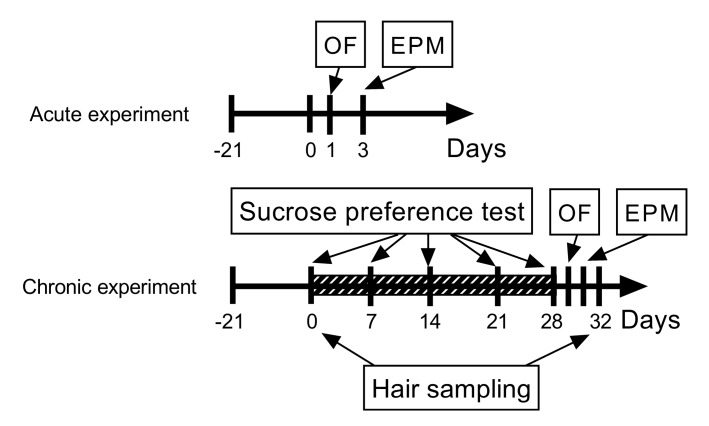
Study timeline to illustrate the design of the acute and chronic experiments, the application of the chronic unpredictable mild stress (CUMS) protocol, and the timing of the behavioral assays and hair sampling. The diagonally striped box shows the 28-day stressing period preceded by a 21-day habituation to single housing. Stressors were applied twice daily starting at 10:00 and 17:00. Cannabidiol was administered in a single dose of 10 mg/kg by intraperitoneal injections in the acute experiment and daily, starting at day 0 until day 32, in the chronic experiment. Abbreviations: OF, open field test; EPM, elevated plus maze test.

Crystalline cannabidiol (99.5% purity from Trigal Pharma GmbH, Wien, Austria), dissolved in saline containing 4% of dimethyl sulfoxide (DMSO, Sigma-Aldrich, St. Louis, MO, USA) and 1% of Polysorbate 80 (Sigma-Aldrich, Steinheim, Germany), was administered to the animals. In the acute experiment, the animals were injected with the vehicle and CBD 1 h before behavioral testing. In the chronic experiment, the same dose of CBD was administered daily at 12:00 by intraperitoneal injection of individually calculated doses, based on the previously measured body weight (10 mg/kg body weight). In the acute experiment, a single dose of CBD was administered, whereas in the chronic experiment, CBD treatment was initiated concurrently with CUMS at day 0. Control groups in both experiments were injected with saline with the same concentrations of DMSO and Polysorbate 80 in a volume of 1 mL/kg (Figure 1). DMSO was preferred over ethanol as it could be applied in lower concentrations and does not interfere with behavioral assessment [31,32]. Corticosterone reference substance (Cayman Chemicals, Ann Arbor, MI, USA), methanol (Sigma-Aldrich, Steinheim, Germany), acetonitrile (Merck, Darmstadt, Germany), formic acid (Scharlau Chemie, Sentmenat, Spain), and water (Millipore Direct Q10, Merck Millipore, Burlington, MA, USA) used for analytical procedures were of HPLC grade.

### 2.3. Chronic Unpredictable Mild Stress (CUMS)

The rats included in the chronic experiment were chronically exposed to various randomly scheduled, low-intensity, social and environmental stressors throughout the four weeks of the procedure, based on a previous study [33]. These stressors consist of cage tilting (45°, for 24 h), damp bedding (250 mL water per cage, for 24 h), removal of sawdust (for 24 h), cage swap (for 24 h), introduction of a foreign object, water jet (5 mL of 4 °C water suddenly poured on the head of the animals), paired housing (two animals in each cage for a 7 h period; each rat was paired with a different rat each time), strobe flash (14 Hz intermittent light for 1 min), hot-air steam (from a hairdryer for 10 min), and deprivation of food or water (for 24 h). Two of these 11 stressors were applied each day as indicated in Table 1.

### 2.4. Behavioral Assays

The most frequently used outcome measure of anhedonia is the sucrose preference test (SPT), and CUMS has been proved to decreased sucrose consumption. However, it might be unreliable, especially when it is applied to “normal” rats [34]. For the assessment of the behavioral aspects, the open field and elevated plus maze tests were performed at days 1 and 3 in the acute experiment and at days 29 and 31 in the chronic experiment to measure anxiety-like behavior and exploratory and locomotor activity (Figure 1).

#### 2.4.1. Sucrose Preference Test (SPT)

SPT was used to measure the anhedonia-like state on days 0, 7, 14, 21, 28 of the experiment. Rats were provided with two bottles, one containing a 1% sucrose solution, and the other containing water. The bottle order (left-right placement of water vs. sucrose bottles) was counter-balanced among rats in each group to avoid position preference effects. Data were collected by weighing the bottles before and after the 24 h testing period. Sucrose preference was evaluated as the ratio of consumed sucrose solution to total consumption (preference (%) = sucrose solution intake/total intake × 100). Animals were acclimatized to the 1% sucrose solution on day 0, and those having baseline sucrose preference levels below 60% (n = 1 in the vehicle-treated CUMS group, and n = 4 in the CUMS+CBD group) were regarded as spontaneously anhedonic and were excluded from the study as indicated previously [35,36]. The SPT was only executed in the chronic experiment.

#### 2.4.2. Open Field Test (OF)

To assess the anxiety-like behavior of the animals, the OF test was carried out in a 60 × 60 cm black-based plexiglass box with 50 cm-high transparent walls. The animals were placed in the center of the testing area, then their behavior was recorded in top view for 5 min. The apparatus was disinfected with 70% ethanol after each test. All trials were analyzed with EthoVision XT (Noldus IT, Wageningen, The Netherlands, version 11.5), monitoring the distance moved, the number of entries and the time spent in the center zone (central 30 × 30 cm area), the vertical activity (wall climbing, rearing), and the grooming activity.

#### 2.4.3. Elevated Plus Maze Test (EPM)

For the observation of the exploratory behavior, the EPM test was performed on a plus-shaped device, which comprised two opposite open arms (50 × 10 cm) and two enclosed arms (50 × 10 × 40 cm). The experiments were carried out at 60 cm height from the floor, the rats being placed at the crossroad, facing the open arm [37,38]. The activity of the animals was evaluated based on 5 min recordings. Before each test, the maze was cleaned with 70% ethanol solution. The total distance moved, number of entries and time spent in the open/closed arms, head dipping, rearing, and open-arm preference (ratio of open-arm entries to the total number of entries) were quantified by a computerized analysis system (EthoVision XT, Noldus IT, Wageningen, The Netherlands, version 11.5).

### 2.5. Hair Corticosterone Analysis

**Figure 2:**
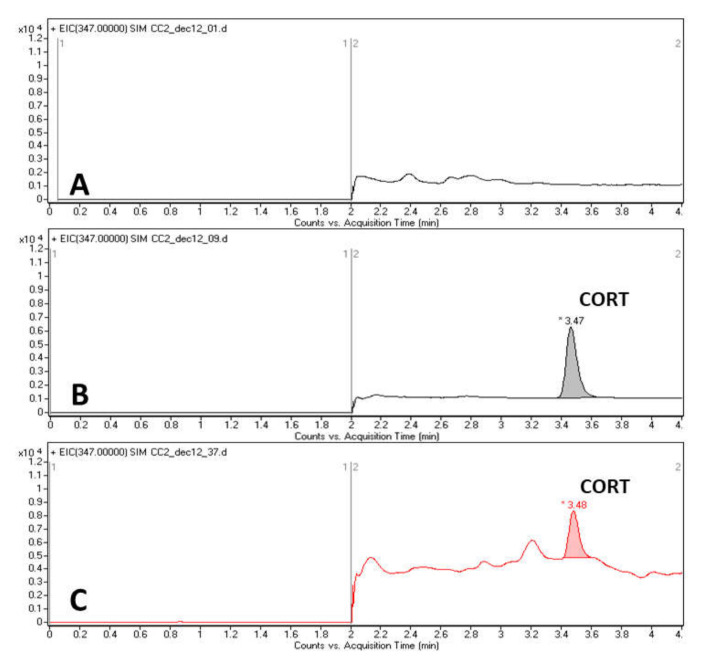
Representative HPLC chromatograms obtained: **A**. blank solution (20 μL 0.1% formic acid (v/v) in deionized water in 150 μL methanol); **B**. standard solution (15 ng/mL), **C**. real sample (10.48 ng/mL).

Hair samples were collected before and after the CUMS procedure for CORT assay. To obtain hair samples without hair follicles, the interscapular area of each animal was shaved three times: on the first day of the habituation (day 21, CORT was not assayed in these samples) and on the first and the last day of the stressing period (day 0 and day 32, respectively). The samples were rinsed two times (2 min) in 2 mL methanol at room temperature and dried for 24 h, then 50–100 mg of the samples was pulverized in a grinding ball mill (UltraTurrax Tube Drive, IKA, Königswinter, Germany). Samples of powdered hair were incubated overnight in 4 mL methanol for CORT extraction. The supernatant was separated, then evaporated to dryness at 37 °C (SpeedVac, Savant, Thermo Fisher, Waltham, MA, USA). The dried residue was reconstituted in methanol (0.2 mL). For precipitation, a volume of 20 μL of 0.1% formic acid (v/v) in deionized water was added to 150 μL of sample solution, then vortex-mixed (1 min) and centrifuged for 10 min at 10,000 rpm (Sigma 2-15 centrifuge, Sigma, Osterode am Harz, Germany). A liquid chromatography–mass spectrometry (LC–electrospray ionization (ESI)-MS/MS) method was developed and implemented for the quantification of CORT, using an Agilent 1100 Series HPLC system coupled with a triple quadrupole mass spectrometer (Agilent Triple Quad G6410A, Agilent Technologies, Santa Clara, CA, USA). The chromatographic separation was performed on a C18 reversed-phase silica gel column (Kinetex Polar C18 100 × 4.6 mm, 2.6 µm, Phenomenex, Torrance, CA, USA) with 0.1% formic acid/acetonitrile (60:40 v/v, 0.6 mL/min flow rate) isocratic elution. The mobile phase was directed to the detector only in the time range of 2.0–4.0 min using a switching valve, including the retention time of CORT (Rt_CORT_ = 3.47 min). At all other times, it was directed to the waste. The evaporation at the ESI interface was performed with a 10 l/min N_2_ gas flow at 350 °C. CORT was quantified without fragmentation, in positive single-ion monitoring (SIM) mode, by detecting the 347 *m*/*z* molecular ion, as published previously [39]. Figure 2 represents the characteristic chromatograms obtained with this method.

### 2.6. Statistical Analysis

The obtained data were evaluated with GraphPad Prism (GraphPad Software, Inc., San Diego, CA, USA, version 5). The Kolmogorov–Smirnov test was performed for normality determination. Quantitative variables are expressed as mean ± SEM unless otherwise stated. Different group sizes are presented for the different tests, since data were excluded from statistical analysis for several reasons. One animal in the control group died at day 3 for unknown reasons. Furthermore, the hair samples of 2 rats in the CUMS+CBD group did not fulfill the different analytical requirements for quantification (e.g., repeatability, sample quality and quantity). Body weight changes, sucrose preference, and hair CORT levels were assessed using two-way repeated-measures analysis of variance (ANOVA) with one between-subjects factor (treatment) and one within-subjects factor of time (day 0–28). Post-hoc comparisons were performed with the Tukey’s multiple comparisons test. Differences with a value of *p* < 0.05 were defined as statistically significant.

## 3. Results

**Figure 3:**
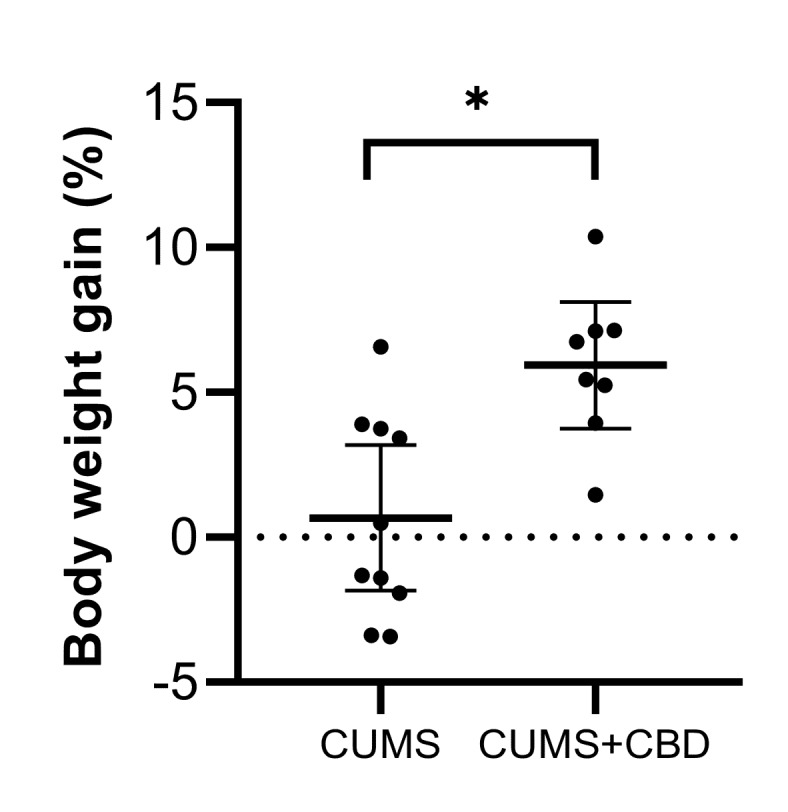
Results of body weight measurements during a four-week CUMS procedure with or without concomitant cannabidiol (10 mg/kg body weight) treatment. Data are expressed as mean ± 95% confidence interval; * *p* < 0.05 vs. control.

Chronic CBD treatment induced an increase of the body weight gain, which was expressed as change (%) to baseline at day 28. Rats in the stressed CUMS group showed a mean (95% CI) weight gain of 0.67% (−1.84 to 3.18) compared to 5.94% (3.75 to 8.13) in the CUMS+CBD-treated group (*p* = 0.0028) (Figure 3).

### 3.1. Sucrose Preference Test

**Figure 4:**
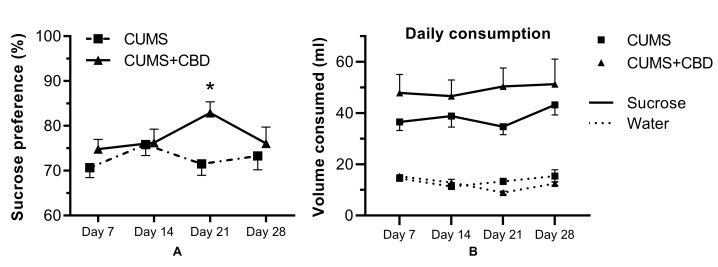
Results of the sucrose preference test for rats submitted to a four-week CUMS treatment with or without cannabidiol administration (mean ± SEM, n = 18). (**A.**) Sucrose preference measured as % preference for sucrose; (**B.**) sucrose (mL of 1.0% sucrose solution ingested) and water intake on each testing day. CBD, cannabidiol; * *p* < 0.05 vs baseline.

The results of the first SPT trial (day 0) were affected by a strong neophobia response to the novel sucrose solution. The second testing (day 7) was considered as the baseline. Compared to this time point, the CUMS group showed no significant modification of the preference for the 1% sucrose solution at days 14, 21, and 28, whereas the CUMS+CBD-treated group had a significant increase in sucrose preference. Repeated-measures ANOVA showed a significant treatment × trial interaction (F(3, 48) = 3.325, *p* = 0.0273), supporting the effect of the CBD treatment that increased sucrose preference to a maximum of 82.9% ± 2.43% at day 21 compared to baseline (*p* = 0.0114); in contrast, the CUMS group showed an insignificant fluctuation between 70% and 75% during the stressing period (Figure 4).

### 3.2. Open Field Test

**Figure 5:**
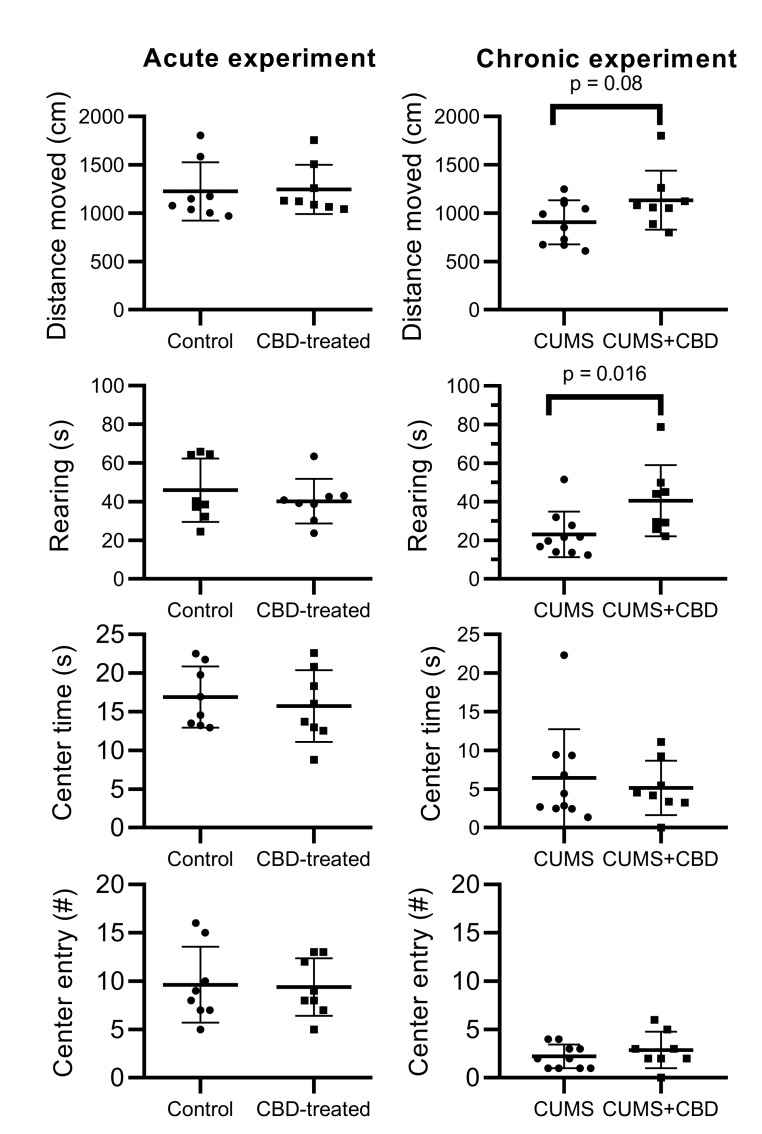
Results of the open field test in the two experiments. The first column represents the acute experiment, where non-stressed animals received a single dose of vehicle or 10 mg/kg of CBD. The second column shows the chronic experiment, where the animals were treated for 32 days with vehicle or 10 mg/kg of CBD and were subjected to the CUMS protocol. Means ± SEM (n = 8–10) are presented. Data were analyzed with unpaired *t*-test or Mann–Whitney test. The dots represent individual values, * *p* < 0.05.

In the acute experiment, the CBD-treated group did not show any significant difference compared to the control group. However, chronic CBD treatment showed significant modifications of the anxiety-like behaviors induced by CUMS. It increased significantly horizontal and vertical exploration, i.e., distance moved, leaning on the walls, rearing, and produced an increasing trend of the number of entries and the time spent in the center zone (Figure 5). Interestingly, CBD-treated animals spent more time grooming than the stressed group. Comparing the acute and chronic effects of CBD, increased locomotor and exploratory activities could be observed in stress-exposed animals only.

### 3.3. Elevated Plus Maze Test

In the acute experiment, the EPM test did not reveal any difference between the groups in the main parameters. In the chronic experiment, both groups showed an anxiety-like behavioral profile in the EPM, exhibiting a low number of entries into the open arms, a short duration of stay in the open arms, and a long duration in the closed arms. There was no significant effect of the CBD treatment on any of the studied parameters; however, the duration of rearing and risk assessment (i.e., stretch attend posture) showed a trend of increase in the CUMS+CBD group (Table 2).

### 3.4. Hair Corticosterone

**Figure 6:**
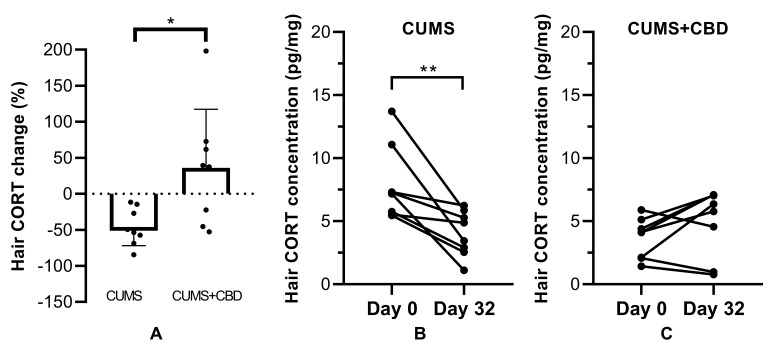
Hair corticosterone (CORT) levels in rats subjected to a four-week CUMS procedure and CBD treatment (10 mg/kg body weight) for 32 days. Samples were taken at day 0 and at day 32 (before and after CUMS). Means ± SEM (n = 8–10 per group) are presented. Data were analyzed by two-way ANOVA with repeated measures for the factors time and treatment. (**A**) Hair corticosterone change expressed as percentage with respect to baseline value; (**B**,**C**) hair corticosterone levels before and after CUMS. The dots represent individual values; * *p* < 0.05, ** *p* < 0.01.

The effect of chronic isolation stress on hair CORT concentration was not significant, as no difference was detectable in drug-naïve non-stressed animals before and after isolation (7.831 ± 2.75 vs 7.501 ± 1.939 pg/mg, *p* = 0.7820). CUMS induced a decrease of hair CORT levels compared to baseline in the stressed group, whereas the CBD treatment slightly increased it (−45.91% vs 36.01%, Figure 6a). Repeated-measures two-way ANOVA analysis detected a significant interaction between CBD treatment and time (F(1, 14) = 16.87, *p* = 0.0011).

## 4. Discussion

The systemic mechanisms by which CUMS-induced HPA activation results in behavioral impairments are now reasonably well understood. The critical factor is that HPA activity is held in check by negative feedback systems operating through forebrain structures, with the primary feedback at the level of the hippocampus [40,41,42]. However, chronic exposure to glucocorticoids is neurotoxic, and hippocampal granule cells are particularly sensitive to these effects, which leads to a loss of the inhibitory effect of the hippocampus on HPA activity.

The endocannabinoid system is known to exert an inhibitory function over the HPA axis, demonstrated by Steiner et al., who observed the elevation of basal and stress-induced CORT secretion in CB1-deficient mice [43]. This regulation and the lately described enhancement of cortical serotonin, glutamate, and gamma-aminobutyric acidergic neurotransmission are the reasons for the endocannabinoid system modulator cannabidiol which increases attention to be considered as a potential therapeutic agent for anxiety and depression [44,45,46,47]. On the other hand, other constituents of the essential oil of *Cannabis sativa*, e.g., terpineol and β-pinene, were also demonstrated to possess antidepressant-like effects [48]. Recently, terpineol was shown to reduce the immobility time in the tail suspension test, an antidepressant-like effect that may involve the cannabinoid receptors [49].

In this study, chronic CBD treatment showed complex behavioral and neuroendocrinological effects in rats exposed to chronic stress. Behavioral despair, anhedonia, and anxiety-like behavior are common signs of depression, which can be identified in rodents as diminished nutrition, disinterest for sucrose or exploration, and reduced tenacity in swimming [24,50]. The absence or improvement of these behavioral patterns, as in our study, can be interpreted as a reduction of stress and prevention of depression. Body weight loss, as an auxiliary indicator of depression was significantly attenuated in the CBD-treated group compared to control rats. This could seem to contradict previous results in which CBD was shown to reduce body weight gain in rats [51] and induce the browning of white adipocytes promoting thermogenesis and lipolysis [52]. However, CBD at 10 mg/kg dose did not decrease the body weight of juvenile rats after a three-week treatment [53]. Moreover, contradicting results were also presented, with CBD increasing the body weight gain in adult rats kept on a high-fat diet in spite of reduced food intake [54]. In this study, the body weight changes of rats that underwent CUMS could reflect the summed impact of stress, food deprivation applied as stressor, and sucrose consumption. It was demonstrated very recently that a 24 h food deprivation may directly affect body weight [55]. Assuming that food access was the same across groups, the body weight gain induced by CBD may be linked to the reduction of stress and to the increase in sucrose consumption. Indeed, long-term sucrose consumption can produce body weight gain in rats also [56]. One of the limitations of this study is the lack of data regarding the impact of the CBD treatment on body weight gain in non-stressed controls.

SPT is a generally accepted measure of anhedonia in rodent depression models; however, inconsistent data were reported in different studies. The majority of publications associate the applied physical and social stressors with a substantial decrease in sucrose solution intake [57,58]. Remus et al. showed that only 35% of the animals had a diminished sucrose preference following a 10-day CUMS protocol, distinguishing stress-resilient and stress-susceptible animals [59]. Similar inhomogeneity in the stress response was described by Strekalova et al. in stress-exposed mice [60]. In order to increase homogeneity within each group, animals showing less than 60% sucrose preference were excluded at the beginning of the study, as described previously [35,36]. After a four-week CUMS procedure, the sucrose preference of CBD-treated rats was significantly higher than that of the controls. In fact, CUMS did not decrease sucrose consumption significantly, the rats still preferring the sucrose solution to water, as reported previously by Murray et al. [34]. However, the increase of sucrose preference in CBD-treated animals can be considered as a prohedonic effect, in accordance with previous results obtained in depressive-like Wistar–Kyoto rats [20].

The behavioral aspects of depression in rodent models and the efficacy of treatment with antidepressants were commonly described by using the forced swim test until recently. The poor construct and face validity of the forced swim test for depression were debated lately, and it is considered more likely that this test measures a behavioral response to an acute inescapable stress rather than a pathological mind state [61,62,63]. On the other hand, the forced swim test should be considered as a high-impact stressor because it was demonstrated to cause thorough changes in HPA axis function and in several neurotransmitter systems (e.g., dopamine, serotonin, gamma-aminobutyric acid) in the brain [62]. Thus, applying the forced swim test at the end of the CUMS procedure may influence the other outcome measures such as CORT levels. Therefore, in this study, the forced swim test was not applied, although most studies which assessed the antidepressant effect of CBD used it [21,64,65]. In the chronic experiment, the open field test revealed an increase in exploratory and locomotor activities in the CBD-treated animals compared to the controls, with most progression in vertical activity (rearing and wall leaning). The differences observed between the acute and the chronic experiments indicate that the CBD treatment affected stress-induced behavioral changes in rats. The non-stressed animals showed increased exploratory and locomotor activities and spent more time in the center zone than any of the animals in the stressed groups. However, the main parameters considered to reflect anxiolytic-like effects remained unchanged following the CBD treatment. Other studies also reported the lack of influence of CBD on the main parameters of the open field test, such as number of square crossings, number of entries, and time spent in the center zone [20,66,67]. On the other hand, vertical exploration (i.e., rearing and wall leaning) by rats was increased by CBD, which should be interpreted with caution. In general, the increase of the number of readings is construed as a reluctance to exploration, but in acutely stressed mice diazepam also induces it [68]. Taken together with the nearly significant increase in horizontal exploration, one may conclude that a chronic CBD treatment lowers the level of anxiety in rats [69]. Conversely, the EPM test did not show any significant difference between CBD-treated and control groups. Similar results were presented by Shoval et al., who also reported that different doses of CBD did not influence the behavioral parameters measured in the elevated plus maze test [20].

Previous studies confirmed that hair CORT determination is a proper noninvasive biomarker for chronic stress assessment in rodents [29,70,71,72], although our study is the first investigating the accumulation of CORT in rat hair, during stress-conditioned CBD treatment. We noticed that four-week chronic stress resulted in reduced CORT accumulation in hair, which could be the result of decreased basal and acute stress-evoked serum CORT levels, as previously reported [28]. Recently, Traslaviña et al. found that HPA axis activity decreases progressively in the CUMS model of depression, but female rats were demonstrated to be more prone to HPA alteration, due to the effect of estrogens [25]. Other studies presented conflicting data. Uarqin et al. reported an increased hair CORT level in rats following long-term overcrowded housing, arguing that social stress induces elevated CORT levels. However, in that study, the decrease of CORT levels was more substantial in the control group than its increase in the overcrowded group [30]. Nevertheless, isolation as well could be a stressful condition; therefore, the verification of its influence on CORT deposition was inevitable. In our study, a negative control group was habituated to handling and kept isolated without additional stress, thus a before–after comparison of CORT levels reflected the effect of single housing. The CORT levels in hair did not change significantly in the negative control group, which augments the accuracy of our data and is in accordance with previous results neglecting the influence of isolation on the adaptation of the HPA axis to chronic stress [73]. Scorrano et al. also found that chronic unpredictable stress increases hair CORT level in rats, reporting a significantly higher CORT concentration in the CUS group than in controls. However, by comparing the CORT levels before and after stressing, it can be stated that the difference between the groups could be rather the consequence of the decreased CORT value in the non-stressed control group [29]. Our findings do not present any reduction of hair CORT in non-stressed controls. In fact, the reason for the dissimilarity could also be the considerably shorter duration of stress exposure in the study conducted by Scorrano et al. compared to ours (9 and 28 days, respectively), presuming that a short-term increase in glucocorticoid concentration is followed by a measurable setback. Steudte et al. came to a similar conclusion examining hair cortisol concentration in patients diagnosed with general anxiety disorder (GAD), which presented severe depressive symptoms. They showed 50–60% lower hair cortisol levels in GAD patients than in healthy controls, assigning this to a compensatory HPA hypofunction [74]. After all, considering that decreased HPA responsiveness is the consequence of prolonged stress-induced activation, the decrease of hair CORT levels in the stressed and non-treated group may be reasonable. The reversal of the CUMS effects on hair CORT indicated a potential anxiolytic or antidepressant effect of the CBD treatment, which clearly needs further confirmation. Taken together, the results presented here and the previous findings in genetic animal models of depression [20,21] support a potential effectiveness of the CBD treatment in anhedonic states.

## 5. Conclusions

CBD exerted a prohedonic effect in rats subjected to CUMS, demonstrated by the increased sucrose preference after three weeks of treatment. The reversal of the effect of CUMS on hair CORT concentrations might also point toward an anxiolytic or antidepressant-like effect of CBD, but this clearly needs further confirmation.

## Figures and Tables

**Table 1:** Chronic mild stress schedule.

Stressors	Days of Experiments
Cage tilting	1, 6, 10, 17, 23, 27, 28
Damp bedding	2, 8, 12, 19, 26
Empty cage	5, 13, 20, 27
Cage swap	18
Foreign object	2, 9, 18
Water jet	12, 16, 22, 25
Paired housing	5, 23
Strobe flashing	3, 6, 11, 17, 20, 22
Hot air steam	4, 11, 16, 24
Food deprivation	6, 13, 20, 27
Water deprivation	4, 9, 15, 24

**Table 2:** Behavioral characteristics of rats in the elevated plus maze test.

Parameter	Acute Experiment			Chronic Experiment		
	Control (n = 8)	CBD Treated (n = 8)	*p*-Value	CUMS (n = 10)	CUMS + CBD (n = 8)	*p*-Value
Open-arm entries (#)	3.00 ± 0.906	2.75 ± 0.725	0.8326	2.30 ± 0.423	2.13 ± 0.295	0.7515
Open-arm preference (%)	25.84 (0.00–38.46)	27.21 (0.00–66.67)	0.9587	12.92 (10.0–22.04)	10.82 (9.64–18.26)	0.3714
Open-arm time (s)	17.12 ± 4.82	22.51 ± 7.72	0.5636	26.42 ± 6.616	27.27 ± 5.963	0.9287
Closed-arm time (s)	200.9 ± 13.51	193.3 ± 12.44	0.6840	196.0 ± 13.79	196.4 ± 10.68	0.9802
Rearing (s)	28.17 ± 3.43	18.55 ± 2.51	0.0401*	21.92 ± 2.056	26.35 ± 2.897	0.1560
Head dips (#)	7.62 ± 2.28	7.75 ± 1.75	0.9660	5.50 ± 0.833	5.38 ± 0.905	0.9206
Distance moved (cm)	1021 ± 109.3	1014 ± 97.56	0.9609	973.1 ± 112.7	916.5 ± 79.33	0.7635
Stretch attend posture (s)	54.50 ±8.20	71.86 ± 10.14	0.2044	25.77 ± 4.66	39.22 ± 7.28	0.1255
Velocity in open arm (cm/s)	4.16 ± 0.72	4.59 ± 1.11	0.7515	3.83 ± 0.439	4.92 ± 0.403	0.0907

* Statistically significant results. Data are expressed as mean ± SEM or median (range) for parametric and non-parametric data, respectively.
